# A Case of Laparotomy, &c.

**Published:** 1889-06

**Authors:** X. O. Werder


### A CASE OF LAPAROTIMY FOR EXTRA-UTERINE PREGNANCY.

(Read at the Allegheny County Med. Society, March 19, 1889.)

BY X. O. WERDER, M. D.,

At the November meeting of this Society I reported a case of extra-uterine pregnancy in which I had performed abdominal section with a successful result. To-day I present the specimen of my second case of tubal pregnancy, removed by laparotomy, on February 14, of this year.

The history of this case is, briefly, as follows:

Mrs. M., twenty-seven years of age, married, two children, the youngest sixteen months old, has been suffering with periodical attacks of severe abdominal pains for almost a year, for which she several times required medical treatment. During the five or six weeks preceding the operation, these attacks increased in frequency and severity, making her unfit to do her ordinary household duties. Walking almost always produced a great deal of suffering. On January 26, of this year I was consulted for one of these attacks of pain, which was referred to the pelvic region, principally the left side. Making a vaginal examination, I found the uterus enlarged to the size almost of a two-months' pregnancy and to the left of this, in the region of the left tube, a soft, extremely tender mass, which was slightly movable. A careful bi-manual examination could not be made on account of the very great sensitiveness of these parts. She had menstruated regularly every four weeks during the last eight or nine months, and was at this time still nursing her baby. At the two subsequent examinations I found no change in her condition, except, perhaps, that this tumor was slightly larger than before. The diagnosis was not quite clear, but I was inclined to the opinion that it was either a hydrosalpinx, or a pyosalpinx, more probably the latter. As her suffering at times had almost become unbearable, I advised laparotomy, to which she readily consented, but the operation was deferred until after her next menstrual period, which was now very close at hand. Menses lasted five days, and presented nothing unusual. In the afternoon of February 13, the day preceding the operation, she came from her home to my office in an open carriage, for the purpose of going to Mercy Hospital. On examination I found her condition unchanged; the mass, however, seemed now decidedly larger. The riding on the rough country road from her home did not seem to have caused her as much suffering as expected, and she was cheerful, and feeling better than for several days previously. But on her way to the hospital the pains returned in unusual severity, and she arrived there faint and nearly collapsed. Several hypodermic injections of morphia made her more comfortable, but she continued very sick and sore all night. On my morning visit before the operation she looked very pale, and was very feeble, still suffering considerable pain. Vaginal examination was not made.

On opening the peritoneal cavity dark blood escaped from the wound, and the abdomen was found containing a considerable quantity of blood, liquid and coagulated. In reaching down for the sac, on the left of the uterus, I felt a small rent in it, probably one-half inch long, which, however, in trying to bring it to the surface, was enlarged, so that all its contents escaped into the abdominal cavity. The bleeding was now very free, the blood being bright red, and easily distinguishable from the old dark blood already contained in the abdomen. The sac was now tied off, and the clots contained within the pelvic cavity turned out. After washing out the abdomen with hot distilled water, it was closed, leaving, however, a glass drainage tube. Blood continued to discharge from this tube for three days, when it was removed.

The patient rallied very nicely from the operation, and made an uninterupted recovery, her temperature and pulse remaining perfectly normal after the fourth day. She left the hospital on the twenty-first day, and is now in good health.

Rupture of the tube must have taken place on her way to or at the hospital, probably as a consequence of the jolting of the carriage. This evidently was the cause of the faintness and slight collapse after her arrival at the hospital the evening before the operation.

This case illustrates again the great difficulties in diagnosing extra-uterine pregnancy, and I cannot agree with Hanks, when he states that the diagnosis can be made in at least 95 per cent. of cases. The case also demonstrates that this interesting affection is by no means such a very rare condition as some seem to suppose, as this is the second case occurring in my own practice within the period of four months. That this was a case of tubal pregnancy there could be very little doubt, but in order to be perfectly certain, I sent the specimen to Dr. William H. Welsh, of Johns Hopkins University, Baltimore, for examination, and he verified the diagnosis. The specimen, he states, consists of an ovary, part of a Fallopian tube, the intervening broad ligament, the fœtal membranes and a placenta with umbilical cord.—*Med. Times and Register.*

